# VPExam Virtual Care for Heart Failure Optimizing Transitions of Care Quality Improvement Project (VPExam QI)

**DOI:** 10.1016/j.cvdhj.2022.04.002

**Published:** 2022-05-11

**Authors:** Nischay Shah, Anthony Annam, Nickolas Cireddu, James V. Cireddu

**Affiliations:** ∗ University Hospitals Regional Medical Centers, Bedford and Richmond Heights, Ohio; † Hondros College of Nursing, Independence, Ohio; ‡ University Hospitals Harrington Heart and Vascular, Cleveland, Ohio; § Case Western Reserve University, Cleveland, Ohio

**Keywords:** Telemedicine, Quality improvement, Remote patient monitoring (RPM), Transition of care, Physical exam

**Key Findings**

• A total of 84% of VPExam QI patients required moderate-significance modification of clinical care based on VPExam data, including volume status assessment with jugular venous pressure (JVP)/edema (47.6%), cardiopulmonary auscultation (33.3%), electrocardiogram detection of arrhythmias (14.2%), and structured data transmission of vitals, medication reconciliation, and labs (95.2%).
• Nursing satisfaction (4.8/5) and compliance with follow-up (100%) was high. No significant technical errors were detected with deployment on over 550 devices.
• VPExam QI intervention was associated with a 30-day hospital readmission rate of 9.52% from a baseline readmission rate of 15.9%, with a relative risk reduction 40.16%. Readmission rates fell precipitously, to 0%, for the last 4 consecutive months of VPExam QI. Thirty-day mortality rate was 4.76%.
• VPExam QI provides supporting quality and feasibility data for increasing adoption of virtual care platforms with minimal barriers. Actionable physical exam and structured data optimize quality of transitional care, allowing for significant reductions in readmissions and mortality.

## Introduction

### Challenges in heart failure

Heart failure (HF) is a leading cause of morbidity and mortality in nearly 5.7 million Americans. With improved survival and an aging patient population, the cost associated with HF management is expected to reach close to $70 billion by 2030.^1^ Skilled nursing facilities (SNFs) are progressively used to care for older patients with significant comorbidities, including HF.^2^ However, morbidity and mortality rates are high for hospitalized HF patients discharged to SNFs, with 30-day readmission rates between 27% and 43%, owing to errors in transitions, inadequate discharge planning, and lack of appropriate follow-up with health care providers.^3^ Optimizing HF management in SNF populations who are underserved yet at high risk for hospitalization is a critical area of focus for value-based care.

The COVID-19 pandemic has increased utilization of telemedicine for HF management. Telehealth is a growing field of importance owing to value-based care and population management incentives, as well as increased acceptance in the general and medical population since the COVID crisis. Numerous meta-analyses have demonstrated that compared to conventional care, the addition of telemedicine in HF management leads to reduced hospitalization and mortality.^4^^,^^5^ However, there is a paucity of data regarding the use of virtual care in SNF and home care patients with HF.

Telemedicine platforms have traditionally presented numerous obstacles, including affordability of equipment, difficult-to-use technology requiring extensive training, lack of standardization, and patient privacy issues. These barriers are especially challenging to SNF and home care populations, who often have significant comorbidities, functional impairments, and barriers to transportation and access.^6^

**Figure 1:**
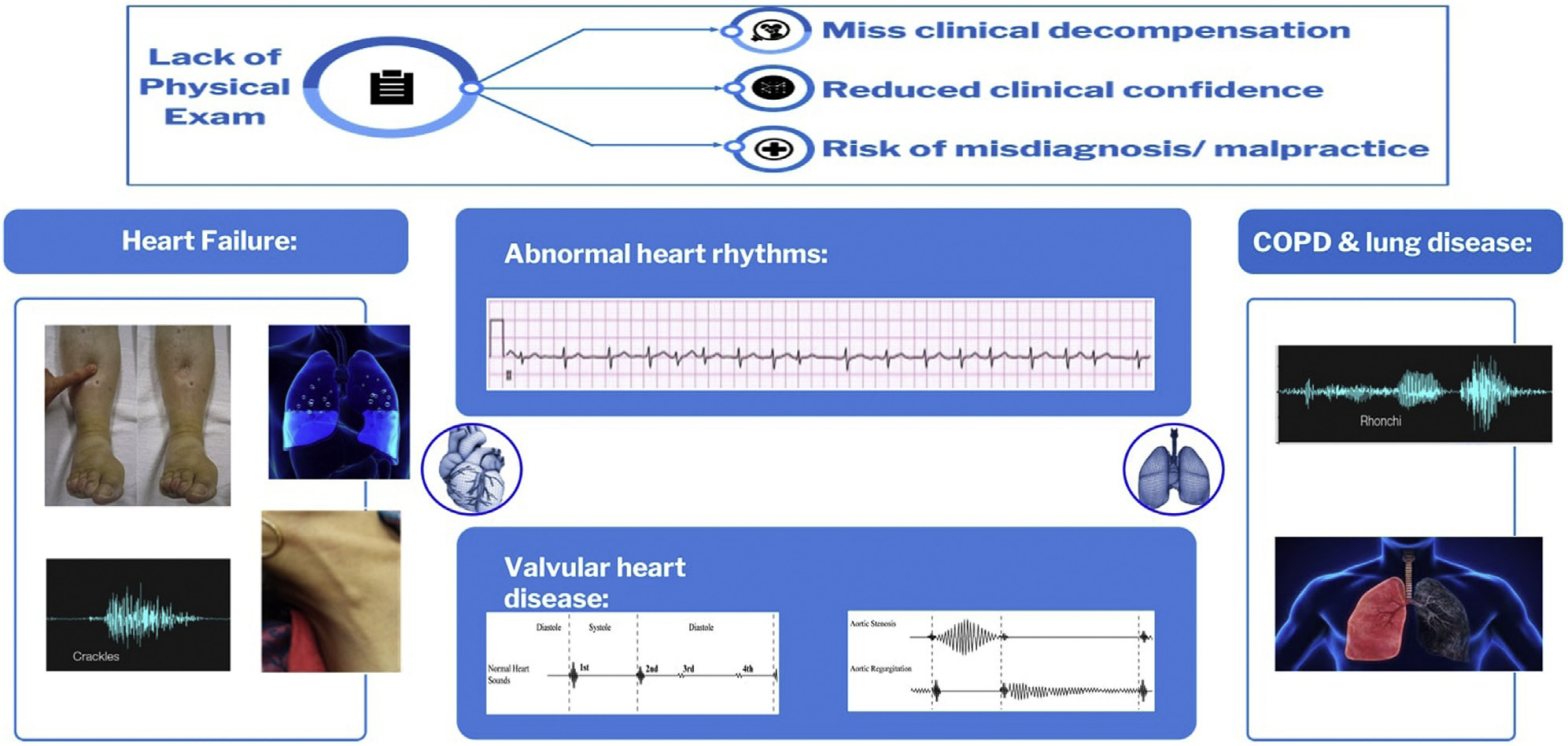
Traditional telemedicine barriers. Lack of physical exam data can result in loss of early enough detection of clinical decompensation, reduced clinical confidence in decision making, and increased risk of misdiagnosis and malpractice. COPD = chronic obstructive pulmonary disease.

Telemedicine can offer these populations improved access, but this is typically at the expense of clinical data such as a comprehensive physical examination and other structured data including vitals, medication reconciliation, and laboratory results. Many of the most common and expensive cardiopulmonary diseases rely on physical examination for detection of decompensation or exacerbation (Figure 1).

### VPExam Virtual Care

VPExam is a Health Insurance Portability and Accountability Act (HIPAA)–compliant medical device data system that assists in overcoming barriers to traditional telemedicine using a combination of augmented reality–based guidance leading a minimally trained nurse or medical assistant (MA) through appropriate camera positioning for video capture based on anatomical landmarks. VPExam augmented reality–guided overlays, combined with sample videos as well as text and audio instructions, teach users how to obtain optimal video recordings of clinical findings such as evaluation of jugular venous distention and degree of lower-extremity edema in a reproducible manner.

**Figure 2:**
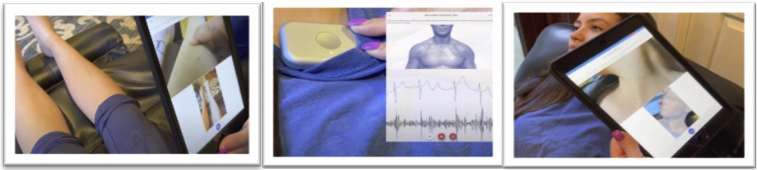
VPExam Clinical App. A combination of augmented reality–based guidance with Bluetooth-enabled stethoscope allows a minimally trained user to capture key physical exam data.

VPExam is integrated with Bluetooth-enabled stethoscopes, including the Eko Duo with single-lead electrocardiogram (ECG), to capture a full heart and lung exam with active user instruction (Figure 2). Virtual physical examination components are customizable by providers to optimize an efficient clinical workflow. The type of physical exam can be customized by medical specialty. Default physical exam components for VPExam QI included evaluation with (1) head-to-toe pan, (2) oral mucosa, (3) jugular venous assessment, (4) lower-extremity edema evaluation, (5) cardiac auscultation with single-lead ECG at the right upper sternal border and right lower sternal border, and (6) pulmonary auscultation at 2 sites.

VPExam app data acquisition creates a partnership between minimally trained users (nurses or MAs) and physicians to efficiently gather and transmit relevant medical data to the Physician Portal. On average, nurses/MAs required a single 20-minute training session before becoming clinically proficient to use the platform in clinical practice. VPExam nurse assessment was completed in under 5 minutes on average. VPExam data were reviewed by the managing physician using the Physician Portal in under 2 minutes on average prior to proceeding to real-time video encounter with the patient and saved in the electronic medical record (EMR). Time-efficient review of asynchronous, clinically relevant patient management data has proven critical for ongoing physician engagement when using virtual care telemedicine.

**Figure 3:**
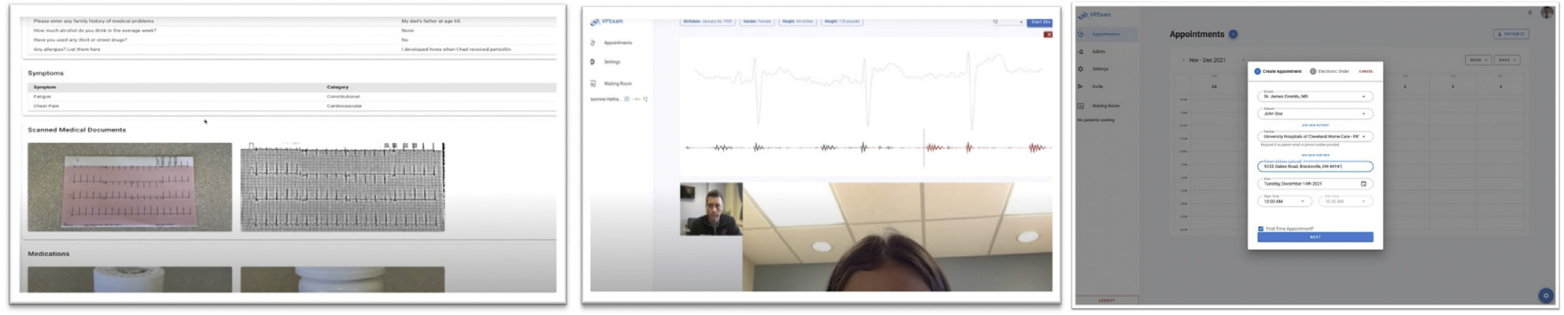
VPExam Provider/Partner Portal. Virtual history, review of systems, medication reconciliation, document scanner, and virtual physical examination are reviewed in the Provider Portal. Skilled nursing facilities and home care receive HIPAA-compliant care coordination orders via the Partner Portal.

In addition to physical exam data, VPExam allows nurses to transmit structured data including manual transmission of vitals, voice recognition–based virtual history and review of systems, medication reconciliation with photographs of pill bottles, and a document scanner to transmit laboratory results, orders, logs, ECGs, etc (Figure 3). Providers reviewed VPExam Physician Portal asynchronous data prior to initiating a real-time HIPAA-compliant video conference with the patient. The Eko stethoscope with ECG can also be used by the provider during real-time synchronous video conferencing in coordination with the nurse/MA user with the patient (Figure 3).

VPExam routinely transmits digital physical exam data for comprehensive assessment of volume status, cardiopulmonary auscultation, ECG detection of arrhythmias, and structured data including vitals, medication reconciliation, and labs. VPExam Partner Portal allows physicians to collaborate with partners at SNFs and home care to schedule VPExam follow-up post discharge. VPExam Partner Portal sends automated e-mail notifications to administrators or directors of nursing at partner facilities. SNFs and home care have HIPAA-compliant access to coordination-of-care data as well as prepopulated electronic home care or SNF orders sent by physician users (Figure 3).

**Figure 4:**
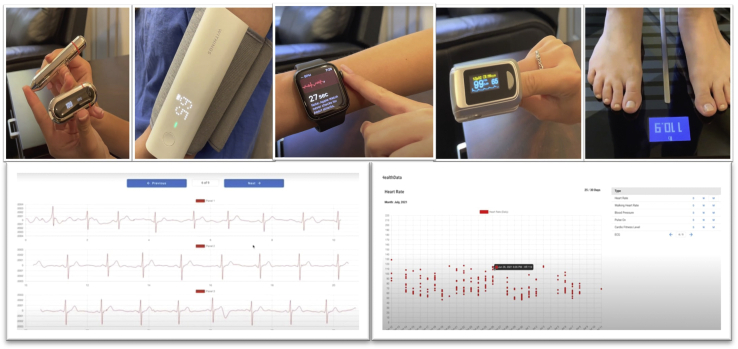
VPExam remote patient monitoring. Smartwatch data including electrocardiogram, heart rate, and pulse oximetry, as well as blood pressure, blood glucose, and scale data, can also be transmitted to the Provider Portal for earlier detection of decompensation.

VPExam also offers additional remote patient monitoring (RPM) using Apple Health and Google Fit Health Kit data transmission to the VPExam Provider Portal, including blood glucose, body mass index, cardio fitness, blood pressure, ECG, heart rate, oxygen saturation, walking heart rate, and weight (Figure 4). VPExam RPM allows for transmitting alerts such as tachycardia with arrhythmia from a smartwatch, revealing a patient going into atrial fibrillation, or greater than 3-pound weight gain from a Bluetooth scale, revealing a patient suffering from decompensation of HF. Abnormal RPM data can trigger deployment of personnel to perform VPExam in a patient’s home or facility for earlier intervention by a physician. For VPExam QI, supplemental health kit–based RPM hardware was not routinely deployed as part of the workflow. The focus was placed on the core VPExam technology of a tablet paired with Bluetooth stethoscope to transmit physical exam data as the primary intervention in order to avoid additional confounders.

## Methods

VPExam QI was designed as a single-arm prospective comparative community case study recruiting patients admitted for HF at University Hospitals Regional Medical Centers with anticipated discharge to SNFs or home care as well as those requiring urgent cardiology consultation with VPExam intervention. Primary outcomes included the degree to which unique VPExam digital physical exam data to assess volume status, cardiopulmonary auscultation, ECG detection of arrhythmias, and structured data of vitals, medication reconciliation, and labs would impact individual transition-of-care management as well as VPExam impact 30-day all-cause rehospitalization rates. The design also sought to assess feasibility of transition-of-care workflows within SNFs and home care, compliance with follow-up, technical stability of the platform, nursing staff satisfaction with the platform, and financial analysis of virtual care networks.

The study design attempted to overcome common barriers to traditional telemedicine. Training barriers were reduced using user-friendly VPExam augmented reality–guided overlays to guide new nurse and MA users through how to obtain optimal video recordings of clinical findings such as jugular venous distention and degree of lower-extremity edema. The VPExam platform was cost-effective, requiring only a tablet and Bluetooth stethoscope at each sending site, allowing for rapid scaling within the health system. VPExam was approved by a thorough institutional Information Technology (IT) Architecture review process by University Hospitals of Cleveland IT Department to ensure HIPAA and HITECH compliance.

### Setting

The goal of VPExam QI was to explore potential clinical benefits to active physician-directed disease management using the VPExam technology and workflows for patients undergoing transitions of care. VPExam QI focused on gathering data on patients being discharged to SNFs or home care from a community-based hospital. VPExam QI evaluated whether a comprehensive virtual care platform can enhance the quality of transitions of care. Three SNF and 1 home care partners were selected with the highest numbers of HF patients being discharged to their care from the 2 medical centers before initiation of the pilot to increase data acquisition. University Hospitals of Cleveland’s IT department successfully deployed VPExam on over 550 hospital-owned devices and University Hospitals home care.

Prior to deployment of VPExam QI, baseline hospital HF outcome data were collected. From June 2020 to June 2021, patients discharged from University Hospitals of Cleveland Regional Medical Centers received guideline-directed HF therapy in accordance with the American College of Cardiology expert consensus guidelines for HF management and follow-up. The same cardiologist and advanced practice providers managed the control population from June 2020 to June 2021, as well as the study population from July 2021 to December 2021, with VPExam acting as the primary intervention.

### Study design

The prospective single-arm community case study of VPExam QI used convenience sampling to recruit patients admitted with HF who met eligibility criteria from University Hospitals of Cleveland Regional Medical Centers. Twenty-one patients with 25 independent patient encounters received virtual care intervention using VPExam technology and established workflows. VPExam QI focused on patients who were being discharged to 3 partner SNFs or follow-up from University Hospitals home care. VPExam QI recruited patients for the 6-month period from July 2021 through December 2021. All patients approached agreed to participate. Once patients were enrolled in the VPExam QI, encounters were completed as routine follow-up appointments within 1–3 weeks of discharge. Partners were also permitted to enroll urgent encounters as part of VPExam QI in coordination with the research team to allow for urgent cardiology consultation.

The intervention of VPExam was compared to historical community matched controls. The control group data were derived from historical community patient data from the hospital system in the months and year immediately prior to VPExam intervention. The control group received guideline-directed HF therapy without routine follow-up at their SNF or home care visits with cardiology-led telemedicine with physical exam data from the same cardiology team as the intervention group.

### Inclusion/exclusion criteria

To identify potential patients, a daily automated list was generated from the EMR of patients admitted to the 2 hospitals for HF, known as the “Currently Admitted Patients with Heart Failure” from Allscripts Sunrise EMR.

HF patients were identified based on brain natriuretic peptide (BNP) of greater than 300 or left ventricular ejection fraction (LVEF) of less than 40%. The team emphasized enrollment of patients who had been readmitted to the hospital within the last 30 or 90 days, as well as patients’ being active on cardiology consultation services. The team enrolled patients being discharged to 1 of 3 SNF partners or eligible for University Hospital Home Health with VPExam capabilities.

Exclusion criteria included the following: (1) those not meeting the definition of HF patients used in VPExam QI; (2) severe dementia preventing the patient’s participation; (3) patients who are unable or unlikely to comply with telemedicine encounters.

### Objective and outcome measures

VPExam QI’s primary objective was to determine the degree of significance of physician-directed disease management based on unique VPExam data including volume status assessment, cardiopulmonary auscultation, ECG arrhythmia detection, and structured data transmission, as well as the downstream impact on 30-day all-cause rehospitalization rates.

Outcome measures included the following: (1) identifying the degree of significance of disease-altering care (minor, moderate, or major) based on VPExam virtual care data (Table 1); (2) 30-day all-cause hospitalization rates; (3) compliance with follow-up visits post discharge; (4) nursing staff satisfaction with using the VPExam platform using a 6-point Likert scale (0 being completely dissatisfied to 5 being completely satisfied); (5) evaluation for technical errors of software using a 6-point Likert scale (0 meaning no technical error and 5 meaning unable to complete telemedicine encounter owing to technical error); and (6) 30- and 90-day all-cause mortality rates.

**Table 1:** Significance criteria for modification of clinical care

Modification of clinical care	Example
Minor or no change in medical care	Stable patients with benign VPExam data without need to alter planned therapy
Moderate changes in medical care	VPExam data result in diuretic and vasoactive medication adjustments or detecting errors in medication reconciliation
Emergent changes in medical care	VPExam used emergently to make decisions whether to triage a patient to the emergency room

With each encounter, demographic data points were collected including sex, body mass index, tobacco abuse history, Charlson Comorbidity Index, NYHA classification, days since previous hospitalization, relevant echocardiogram data with LVEF, degree of valvular disease and pulmonary hypertension, and laboratory data including BNP and glomerular filtration rate trends (Table 2).

**Table 2:** Demographic and clinical characteristics of patient participants

Characteristics	VPExam intervention group
Age, mean (SD), years	79.2 (13.4)
Sex, n (%)	
Male	13 (59.0)
Female	9 (40.9)
BMI, mean (SD)	30.5 (9.4)
Smoking, n (%)	
Yes	2 (9.0)
No	20 (90.9)
Charlson Comorbidity Index, mean (SD)	8.0 (2.5)
Dementia, n (%)	
Yes	5 (22.7)
No	17 (77.2)
NYHA class, n (%)	
I	0 (0.0)
II	11 (50.0)
III	11 (50.0)
IV	0 (0.0)
LVEF,† mean % (SD)	35.9 (15.2)
BNP,† mean (SD)	1069.3 (1500.9)
GFR on discharge from hospital,† mean (SD)	40.5 (23.5)
Significance of modification to clinical care, n (%)	
Minor	4 (15.3)
Moderate	21 (84.6)
Diuretic adjustment	11 (44)
Vasoactive adjustment	11 (44)
Medication reconciliation error	4 (16)
Antiarrhythmic adjustment	2 (8)
Emergent	0 (0)
VPExam data linked to modification of care, n (%)	
Volume status evaluation (JVP and edema)	10 (47.6)
Auscultation capability (wheeze, rhonchi, murmur)	7 (33.3)
ECG capability (AF detection)	3 (14.2)
Structured data (vitals, medical records, lab transmitted)	20 (95.2)
Use of guideline-directed therapy, n (%)	
Beta blocker	20 (95.2)
Renin-angiotensin inhibitor	8 (38.0)
Mineralocorticoid receptor antagonists	2 (9.5)
Nursing satisfaction (0–5), mean (SD)	4.84 (0.22)
Technical error (0–5), mean (SD)	0 (0)
Hospital admission, all-cause, n (%)	
30-day	2 (9.52)
Mortality, all-cause, n (%)	
30-day	1 (4.76)
90-day	4 (19.04)

AF = atrial fibrillation; BMI = body mass index; BNP = brain natriuretic peptide; ECG = electrocardiogram; GFR = glomerular filtration rate; JVP = jugular venous pressure; LVEF = left ventricular ejection fraction.

† Data based on 21 participants.

### Data analysis

Demographic and functional characteristics were measured using descriptive statistics. Continuous variables were expressed as mean ± standard deviation if normally distributed, or otherwise as median (interquartile range).

### Ethics

The intervention was implemented as a virtual care practice change to improve the quality of care. Data were collected and stored using a HIPAA-compliant, web-based data collection tool stored in a restricted-access folder on a secure server. All participants received information about the purpose of the study, agreed to consent before participation, and were free to withdraw from the study at any time. Informed consent was obtained from the individuals or from minors’ legal guardian/next of kin for the publication of any potentially identifiable images or data included in this article.

## Results

### Study population

The community case study recruited 21 patient participants with 25 independent encounters for patients discharged to SNFs or home care from 2 community hospitals, University Hospitals of Cleveland Regional Medicals Centers. At baseline, patients were on average 79.2 (SD 13.4) years old (Table 2), 40.9% of the population was female, and only 9.0% were active smokers. The average body mass index of the study population was 29.6 (± 9.4). BNP on admission was on average 1069.3 (± 150.9). The average glomerular filtration rate on discharge for patients was 45.4 (± 23.5). The mean LVEF was 35.9% (± 15%). Of those patients, 41.6% had HF with reduced LVEF and 58.3% had HF with preserved LVEF. Participants were found to be in NYHA class II (50.0%) or NYHA class III (50.0%). The mean Charlson Comorbidity Index was 8.0 (± 2.5), reflecting a cohort with significant comorbidities and an extremely poor (0%) estimated 10-year survival.

Nursing satisfaction overall rating of using this platform was 4.8/5 (± 0.22) for 25 surveys of nursing staff that participated. Staff were also interviewed regarding technical difficulty encountered with the use of the platform on a 6-point Likert scale, with 0 meaning no technical error and 5 meaning unable to use the platform owing to technical error. For all nursing staff involved in the study, the score was zero. No technical errors were detected across partners at 3 SNFs and deployment on over 550 home care–owned medical devices.

VPExam QI patients received clinical follow-up for approximately 2–3 weeks’ duration and data collection for 3 months following VPExam encounter. There were no patients who declined participation and there were no dropouts. All 21 patients and 25 scheduled encounters at partner SNFs and home care nurses were compliant with VPExam visits coordinated by communication via VPExam Partner Portal. A total of 19% of study encounters occurred with home care services and 81% of encounters occurred at SNFs. During the 6-month pilot, 33.8% of all hospitalized HF patients at University Hospitals of Cleveland Regional Hospitals were enrolled into VPExam QI. Cardiology management was based on guideline-directed therapy. Over 95% of patients were on beta-blocker therapy. A total of 38% of patients were on renin-angiotensin inhibitors and 9.5% of patients were on mineralocorticoid receptor antagonists on follow-up visits during the study. The most common documented reason for deferring renin-angiotensin inhibitors or mineralocorticoid receptor antagonists was acute renal failure, followed by allergy, including angioedema, followed by hyperkalemia.

**Figure 5:**
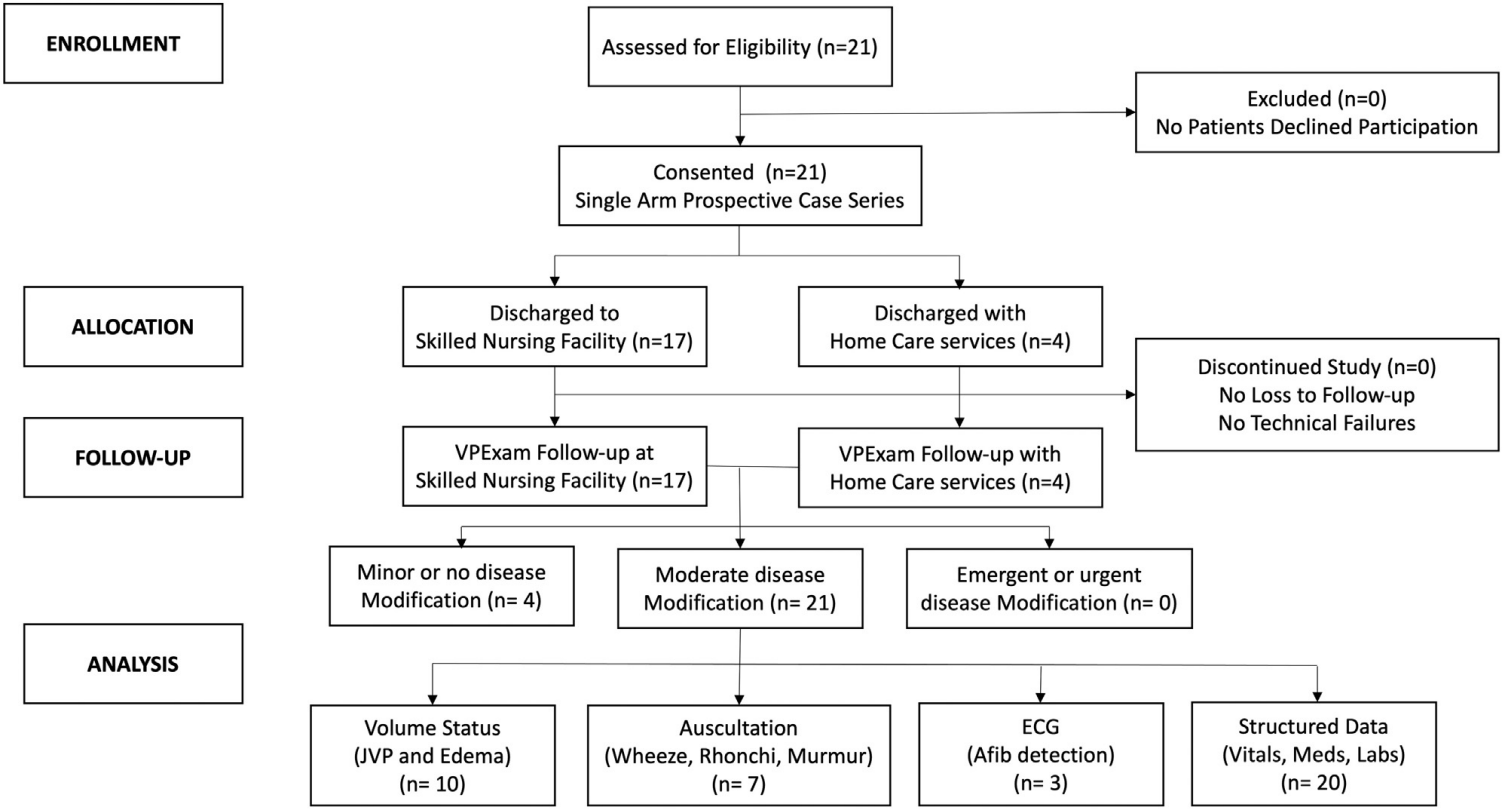
Patient participation flow chart. A total of 84% of VPExam QI patients required moderate-significance modification of clinical care, improved by volume status assessment including jugular venous pressure (JVP) and edema (47.6%), cardiopulmonary auscultation (33.3%), electrocardiogram (ECG) (14.2%), and structured data transmission (95.2%).

Sixteen percent of patient encounters required only mild or less significant clinical modification of care, while 84% of patient encounters required moderate-significance changes in clinical modification based upon VPExam follow-up (Table 2, Figure 5). The most common moderate-significance changes involved diuretic adjustments (44% of encounters) and vasoactive medication adjustments (44% of encounters). Less frequent moderate-significance clinical modifications included identification of medication reconciliation errors in 8% of encounters and alteration of antiarrhythmic medications in 4% of encounters. Moderate-significance modification encounters were impacted by volume status assessment (47.6%), cardiopulmonary auscultation (33.3%), ECG detection of arrhythmias/atrial fibrillation (14.2%), and structured data transmission of vitals, medication reconciliation, and lab results (95.2%). Nursing satisfaction with the platform was very high.

Prior to deployment of VPExam QI, baseline hospital HF outcome data were collected. From June 2020 to June 2021, patients discharged from University Hospitals of Cleveland Regional Medical Centers received guideline-directed HF therapy in accordance with the American College of Cardiology expert consensus guidelines involving medical/procedural management and follow-up. The same cardiologist and advanced practice providers managed the control population from June 2020 to June 2021, as well as the intervention population from July 2021 to December 2021, with VPExam acting as the primary intervention.

**Figure 6:**
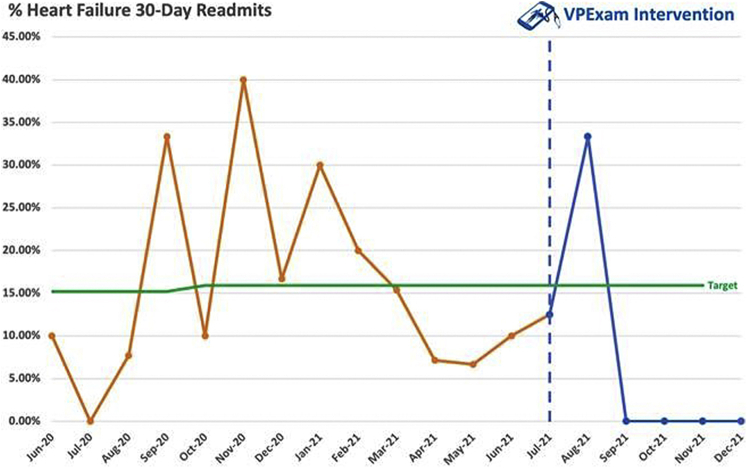
Hospital readmission rates before and after VPExam QI intervention. Hospital readmission data have an average monthly 30-day readmission rate of 15.91% for the year leading up to VPExam QI. During the initial 6 months of VPExam QI, the average monthly 30-day readmission rate for all University Hospital of Cleveland Regional Hospitals fell to 7.98%. There were 0% readmissions found for the last 4 consecutive months of VPExam QI.

The historical control population of HF patients discharged from University Hospitals of Cleveland Regional Medical Centers from June 2020 to June 2021 had an average monthly all-cause readmission rate of 15.91%, with 158 total encounters. Following intervention with VPExam QI interventions, 30-day hospital admission rates dropped to 7.98% for the entire HF population, with 62 total encounters. Within the 21 encounters of VPExam QI, the all-cause 30-day hospital admission rate was 9.52%. VPExam was associated with a 40.1% relative risk reduction for 30-day readmission compared to historical community matched control. Of note, fewer than 25% of VPExam QI patients were recruited in months 1 and 2 of the pilot when the readmission rate for the hospital system remained similar to historical averages. However, by months 3–6 of the pilot more than 75% of VPExam patients were recruited and readmission rates fell precipitously to 0% for 4 consistent months (Figure 6).

Across HF subtypes, patients with HF mortality rate following hospitalization is approximately 10.4% at 30 days, 22% at 1 year, and 42.3% at 5 years.^7^ Thirty-day mortality in VPExam QI was significantly lower at 30 days, at 4.54%, with a relative risk reduction of 56.3% compared to historical controls. Of note, 90-day mortality of the study population was significantly higher at 19%, reflecting a patient population that was high risk given significant comorbidities, as reflected by an average Charlson Comorbidity Index of 8.

The VPExam QI used the ongoing results for a rapid-cycle improvement strategy via Plan-Do-Study-Act cycles. Continually implementing improvements enables providers to better serve the highest-risk patients and improve quality for transitions of care. Plan-Do-Study-Act cycles also help hospital systems overcome resistance to new virtual-care technology workflows.

## Discussion

### Barriers to quality improvement

Significant barriers identified in literature review preventing successful integration of quality improvement initiatives include the following: (1) the presentation of HF can be nonspecific and make it difficult to diagnose; (2) guideline-directed drugs are underused owing to doubt on their utility and safety concerns; (3) SNF providers can be uncomfortable managing HF owing to complexity and autonomy at end-of-life decisions; (4) there is mistrust and lack of timely communication between SNF staff, patients, and their families; (5) there are limited resources in terms of diagnostic studies and access to specialists for care of patients; and (6) there are poor information sharing and transfer capabilities.^2^^,^^6^^,^^8^^,^^9^

VPExam QI reveals that unique VPExam tools overcome many of these barriers while creating a successful local virtual care network between hospital systems and partner SNFs and home care.

### VPExam feasibility

VPExam deployment to SNFs and home care is feasible, scalable, and cost-effective. VPExam virtual care networks enable high compliance with follow-up, with high satisfaction from nursing staff. A major value for moderate-level-complexity patient management involved structured data transmission of the platform linked to moderate modification in clinical management over 95% of encounters. Structured data transmission of vitals improved detection of uncontrolled hypertension, hypotensive episodes, and tachyarrhythmias often missed in traditional telemedicine. Structured data transmission of medication reconciliation often identified high-risk transition-of-care errors. Structured data transmission of labs including renal function, BNP trends, and blood count stability improved the quality of medical decision making.

VPExam is also uniquely suited to improve clinical decisions with unique physical exam data. Volume status including jugular venous distention and lower-extremity edema influenced moderate modification management in 50% of encounters. Synchronous and asynchronous stethoscope auscultation of the heart to detect murmurs, rubs, gallops, and irregularity, as well as auscultation of the lungs to detect wheezing and rhonchi, influenced moderate modification management in over 31% of encounters. Synchronous and asynchronous single-lead ECG transmission for arrhythmia detection including atrial fibrillation influenced moderate modification management in over 13.6% of encounters.

The results demonstrate that virtual care in transitions of care to SNFs and home care offer a large opportunity to improve quality of care and mitigate risk of errors for cardiopulmonary patients. VPExam was associated with improved adjustments in medication dosages based on physical exam data, correcting errors in medication reconciliation, and educational reinforcement of medication compliance. Despite a relatively small cohort, the reduction in 30-day hospitalization and mortality supports the hypothesis that unique VPExam virtual care data empower care providers to optimize transitional quality of care beyond traditional guideline-directed therapy.

### VPExam virtual care financial analysis and feasibility

Beyond quality improvement encouraging adoption of virtual care, there are also financial considerations for various stakeholders. Transfer of SNF residents to emergency departments is linked to increased morbidity and mortality, as well as significant cost.^8^ The average cost of readmission is $15,200 across various payers. Moderate-significance VPExam clinical modification is associated with a significant reduction in 30-day hospitalizations. Within cardiology, there are over 233,100 readmissions for HF, 81,600 readmissions for cardiac arrhythmias, and 74,300 readmissions following myocardial infarct annually. Other high-risk conditions with benefit from VPExam cardiopulmonary and volume status evaluation include chronic obstructive pulmonary disease with 106,300 readmissions, pneumonia with 7,500 readmissions, and renal failure with 96,900 readmissions annually.^10^

VPExam helps reduce the cost of transporting patients to specialists and hospitals, including ambulance cost and nursing time expenses. Virtual care also helps reduce the risk of contracting infectious diseases such as COVID while visiting the health care facility, as well as the unforeseen burden to the patient, including potential need for quarantine.

In addition to the financial incentivization to using virtual care to mitigate the cost of readmissions, the platform can also increase individual provider productivity. VPExam data were correlated to a higher level of complexity per encounter on average, increasing CPT 99454 to CPT 99457 with an average increase in reimbursement of approximately $50 and 0.9 work relative value units (wRVU). Furthermore, VPExam services are eligible for RPM billing using CPT codes 99453, 99454, 99457, and 99458 with recurring monthly payments. VPExam QI service received RPM reimbursement from diverse payors, including Medicare A, United Healthcare Medicare HMO, Humana Gold Choice, CareSource MyCare Ohio, and United Healthcare Medicare HMO, throughout the pilot.

VPExam QI is a quality and feasibility study during an unprecedented COVID crisis. We suspect that launching similar transition of virtual care programs in other hospital systems at a larger scale as a formalized quality initiative will be beneficial to ensuring high-quality outcomes across the continuum of care. Virtual care offers earlier detection of clinical decompensation to underserved communities and better decisions by clinicians, resulting in reduced risk of hospital admission and mortality on discharge. In turn, improved transitional care assists health care systems in preventable penalties, unnecessary care escalation, and more efficient utilization of limited inpatient and intensive care unit beds.

### Study limitations

A significant limitation of the VPExam QI is the relatively small scale of a 21-patient single-center observational trial owing to the resources needed to deploy technology, train staff, and allow for adequate data collection in a relatively condensed timeline. This made a case series study with descriptive statistics the most appropriate format for analysis. The interventions were deployed from community-based hospitals in suburban areas that may not be applicable to more urban or rural health care settings. Scalability may be resource constrained by access to technology, especially for smaller health systems. Partnership for VPExam technology deployment was made with 3 high-volume SNFs and a hospital-operated home care agency. Of note, the United States has trended toward decreasing discharges to SNFs in favor of discharging patients home with home care services. Patients discharged to SNFs and home care on average have significantly greater comorbidities and barriers to care than patients discharged to home without such services. Therefore, the intervention of VPExam QI was indirectly focused on the higher-risk HF population.

The research team included 2 board-certified cardiologists, 2 internal medicine residents, 2 cardiology nurse practitioners, and 1 registered nurse. Cardiologists with HF-specific training were available during all VPExam QI encounters to improve standardization of data analysis and guideline-directed therapies. VPExam QI encounters were completed as routine follow-up appointments or urgent consultations. One of the major reasons for a lack of emergent significance modification of clinical management in the VPExam QI was that the small size of the study team made it impossible to provide consistent 24/7 access to cardiology care for urgent decompensations. Therefore, an emergent VPExam workflow was not consistently established with partner SNFs and home care. The value of VPExam technology in these types of emergent encounters can likely be better studied in the future by deploying the platform with a significantly larger clinical team supporting these types of encounters and offers greater potential quality-of-care impact.

### Clinical perspectives

Implementing virtual care to improve transitions of care in the underserved SNF and home care patient populations represents a promising measure to address high readmission rates in HF patients. VPExam offers capabilities to efficiently transmit virtual physical exam cardiopulmonary and volume status assessments with supplemental structured data, including vitals, medication reconciliation, and labs critical to HF management. Noninvasive virtual-care platforms offer novel interventions to optimize quality of care, impacting readmission and mortality rates.

### Translational outlook

Virtual care in HF management is a rapidly evolving intervention with high-yield quality returns for SNF and home care patients, who traditionally have poor access to specialist care. Future projects should not only focus on how virtual care can be integrated to provide better quality of care, but also address the paradigm of cost-effective health care delivery given the growing challenges of health care resource scarcity. Ongoing government and hospital support for digital innovation research allows physicians the opportunity to define the way developing technologies will be used in future workflows to optimize efficiency and quality of care.
